# Integration of Lyoplate Based Flow Cytometry and Computational Analysis for Standardized Immunological Biomarker Discovery

**DOI:** 10.1371/journal.pone.0065485

**Published:** 2013-07-03

**Authors:** Federica Villanova, Paola Di Meglio, Margaret Inokuma, Nima Aghaeepour, Esperanza Perucha, Jennifer Mollon, Laurel Nomura, Maria Hernandez-Fuentes, Andrew Cope, A. Toby Prevost, Susanne Heck, Vernon Maino, Graham Lord, Ryan R. Brinkman, Frank O. Nestle

**Affiliations:** 1 St. John's Institute of Dermatology, King's College London, London, United Kingdom; 2 National Institute for Health Research, Comprehensive Biomedical Research Centre, Guy's & St. Thomas' NHS Foundation Trust, (NIHR GSTT/KCL) London, United Kingdom; 3 Molecular Immunology, National Institute for Medical Research, Mill Hill, London, United Kingdom; 4 Biological Research & Development, BD Biosciences, San Jose, California, United States of America; 5 Terry Fox Laboratory, BC Cancer Agency, Vancouver, BC, Canada; 6 The Medical Research Council (MRC) Centre for Transplantation, King's College London, London, United Kingdom; 7 Division of Genetics and Molecular Medicine, Statistical Genetics Unit, King's College London, London, United Kingdom; 8 Academic Department of Rheumatology, King's College London, London United Kingdom; 9 Department of Primary Care and Public Health Sciences, King's College London, London, United Kingdom; 10 Department of Medical Genetics, University of British Columbia, Vancouver, Canada; La Jolla Institute for Allergy and Immunology, United States of America

## Abstract

Discovery of novel immune biomarkers for monitoring of disease prognosis and response to therapy in immune-mediated inflammatory diseases is an important unmet clinical need. Here, we establish a novel framework for immunological biomarker discovery, comparing a conventional (liquid) flow cytometry platform (CFP) and a unique lyoplate-based flow cytometry platform (LFP) in combination with advanced computational data analysis. We demonstrate that LFP had higher sensitivity compared to CFP, with increased detection of cytokines (IFN-γ and IL-10) and activation markers (Foxp3 and CD25). Fluorescent intensity of cells stained with lyophilized antibodies was increased compared to cells stained with liquid antibodies. LFP, using a plate loader, allowed medium-throughput processing of samples with comparable intra- and inter-assay variability between platforms. Automated computational analysis identified novel immunophenotypes that were not detected with manual analysis. Our results establish a new flow cytometry platform for standardized and rapid immunological biomarker discovery with wide application to immune-mediated diseases.

## Introduction

Chronic inflammation and dysregulated activation of the immune system are central to the pathogenesis of immune-mediated inflammatory diseases (IMID), such as psoriasis, rheumatoid arthritis, and Crohn's disease [1], [2], [3], [4]. However, the precise cellular and molecular mechanisms underlying these conditions are not fully understood. Given the impact on quality of life, productivity, and the high medical costs related to IMID, the need to understand disease immunopathogenesis is accompanied by an urgent demand to identify specific biomarkers for the purpose of disease screening, diagnosis, staging, and monitoring, as well as to evaluate therapy response. A biomarker is a characteristic that is objectively measured and evaluated as an indicator of normal biological processes, pathogenic processes, or pharmacologic responses to a therapeutic intervention [5]. Biomarker discovery is a challenging process; a good biomarker has to be sensitive, specific, and the biomarker test highly standardized and reproducible [6]. High-dimensional flow cytometry has emerged as a suitable tool for the identification of immunological biomarkers, relevant not only for IMID but also for cancer [7], cardiovascular disease [8], allograft rejection and tolerance [9], [10], and infectious diseases [11]. Multicolour flow cytometry has become one of the preferred tools to study the immune system, allowing the simultaneous characterization of many cell types and their functions in complex tissue compartments such as blood, thus opening the way to a faster and more sophisticated biomarker discovery in human immunology. However, currently practised flow cytometry has disadvantages: lack of standardization in reagents and protocols, subjectivity in data analysis, and difficulty to directly compare data from different studies. Thus, there is a clear need for a more sophisticated standardized diagnostic platform.

Human studies are not only challenged by intrinsic human variability, but are also often limited by sample availability. Moreover, such studies are frequently run across multiple centres and over an extended time period. As part of the Federation of Clinical Immunology Societies (FOCIS) Human Immunophenotyping Consortium, we have recently highlighted three main areas impeding the widespread use of flow cytometry in clinical trials: sample handling, instrument setup, and data analysis [12]. Each step, from sample collection to sample processing, storage, and flow cytometry staining, requires harmonized and standardized experimental protocols and reagents in order to obtain reliable results, which can ultimately lead to the identification of robust biomarkers. We have addressed these issues and developed a novel standardized flow cytometry platform.

To simplify and standardize sample and reagent handling, 96 well plate-based assays using lyophilized reagents (lyoplates) for cell stimulation and staining constitute an alternative option to widely used liquid reagents [13]. Lyoplate technology potentially enables reagent standardization, and medium-throughput sample processing, and minimises pipetting errors [14]. However, lyoplate studies to date have only used a limited number of markers and fluorochromes [13], [15], [16], [17].

A further source of variation in flow cytometry is instrument setup prior to sample acquisition. This aspect can be overcome by establishing target values for each fluorescent channel using standard beads [18]. Software for tracking cytometer performance, such as BD Biosciences Cytometer Setup and Tracking or Beckman Coulter MXP, in combination with bead-based standardization are now routinely used to obtain reproducible day-to-day cytometer settings.

Finally, manual analysis of highly multidimensional flow cytometry data is subjective and time consuming. While automated clustering (gating) algorithms have been developed and shown to favourably compare to manual analysis [19], this is only one step in the analytical pipeline. Additional approaches are required that apply the results from the clustering to discover differences and biomarkers in the data set in a more complete fashion than possible via manual gating.

In this study we compared a conventional flow cytometry platform using liquid reagents (CFP) and a lyoplate-based 12 parameter flow cytometry platform (LFP) in a cohort of healthy subjects. We combined an optimised standard operating procedure (SOP) for sample handling, stringent instrument QC, and reproducible instrument settings, followed by advanced computational analysis. Moreover we established an experimental and analytical framework that can be applied to large human studies for immunological biomarker discovery.

We report that LFP showed higher sensitivity to detect key cytokines (IFN-γ and IL-10) and activation markers (Foxp3 and CD25). Moreover, automated computational analysis was able to reliably identify immunophenotypes previously unappreciated via conventional manual analysis.

These results support the integrated use of preformatted lyophilized-reagent plates and computational analysis in flow cytometry experiments as a standardized framework for conducting rapid immunological biomarker discovery in large, human studies.

## Materials and Methods

### Ethics statement

The study was approved by the institutional review board of Guy's Hospital (Guy's Research Ethics Committee, Ethics Committee Code: 06/Q0704/18) and conducted in accordance with the Helsinki Declaration. Written informed consent was obtained from each participant.

### Healthy volunteers

Peripheral blood was collected from 12 healthy volunteers (three males and nine females, mean age 33 years, range 22–51 years) at two time points (four week interval).

### Peripheral blood mononuclear cell isolation and storage

Peripheral blood mononuclear cells (PBMC) were isolated from blood samples of healthy volunteers or from leukocyte cones (National Blood Transfusion Center, United Kingdom), by density centrifugation over Lymphocyte Separation Medium LSM 1077 (PAA Laboratories). Isolated PBMC were frozen in RPMI 1640 (Gibco) containing 11.25% human serum albumin +10% DMSO (Sigma) and stored in liquid nitrogen until use. CD Check plus cells (Streck), a whole blood control for immunophenotyping, were also used.

### Liquid and lyophilized reagents for cell stimulation and flow cytometry staining

Reagents for cell stimulation [Phorbol 12-myristate 13-acetate (PMA), 100 ng/ml; Ionomycin, 1 μg/ml], as well as Golgi inhibitors [Monensin, 5 μg/ml, Brefeldin A (BFA), 5 μg/ml] were all obtained from Sigma; antibodies were obtained from Becton Dickinson (BD Biosciences). The amine-reactive viability dye (LIVE/DEAD Yellow, Life Technologies) was used in the liquid form for both liquid and lyoplate based experiments.

Stimulation plates were formulated by lyophilizing a mix of BFA and Monensin (unstimulated wells) or BFA-Monensin-PMA-Ionomycin (stimulated wells). Staining plates were formulated with antibody mixes containing either anti-human CD3 APC-H7, CD4 APC, CD8 BD Horizon V500, CD45RO PE-Cy5, and CD25 PE-Cy7 (surface stain plates) or anti-human IFN-γ Alexa Fluor 700, IL-10 PE, IL-17A BD Horizon V450 and Foxp3 Alexa Fluor 488 (intracellular stain plates) lyophilized into V-bottom 96-well plates (BD Biosciences).

### Flow cytometry experiments

PBMC were thawed and rested in RPMI 1640+10% FCS +1% penicillin/streptomycin (cRPMI) at 37°C overnight. Next, 1.5×10^6^ cells were incubated either in FACS tubes or wells of the stimulation plate in a final volume of 200 μl of cRPMI for 5 hours at 37°C. Each experimental condition was run in triplicate. Tubes and plates were centrifuged at 300×*g* for 5 min, and supernatants were removed by tube inversion or using a 12-channel manifold (V&P Scientific) for the plates. Cells were stained with the LIVE/DEAD Yellow dye for 20 minutes in the dark. Lyophilized surface stain plate was hydrated with wash buffer (PBS +0.1% NaN_3_ +0.5% BSA), and the liquid or the lyophilized antibody mix was transferred to either tubes or the stimulation plate, mixed and incubated for 30 min in the dark.

Cells were then washed, fixed, and permeabilized with the BD Human FoxP3 Buffer Set (BD Biosciences), according to manufacturer's instructions. Cells were washed twice and the lyophilized intracellular stain plate was hydrated with wash buffer.

The liquid or the lyophilized antibody mix was transferred to either tubes or the stimulation plate, mixed, and incubated for 60 min in the dark.

Cells were washed twice and the samples were acquired on a 5-laser BD SORP (Special Order Research Product) Fortessa in tubes or plates using the High Throughput Sampler (HTS) option within 24 hours of staining.

### Flow cytometer set up and sample acquisition

Experiments were only run when the daily instrument QC using BD CS&T beads had passed. Instrument application settings were created by linking the target values of fluorochromes to a specific CS&T bead lot after ensuring the signal from each fluorochrome was higher than 2.5× rSD and brightest in its own channel. The above assay-specific application settings were used in all the experiments. Gates were set using Fluorescence Minus One controls and unstimulated samples for cytokines. Acquisition stopping gate was set at 40, 000 live CD4^+^ T cells, and samples not reaching this stopping gate were excluded from the analysis.

### Data analysis and statistics

Manual flow cytometry data analysis was done with FlowJo (TreeStar) or FACSDiva (BD Biosciences). Specific cell frequencies obtained from each donor were averaged by experimental triplicates and the agreement between the frequencies obtained with the two experimental methods was assessed using the Bland-Altman 95% limits of agreement. Systematic differences between the frequencies from the two methods were analysed using the paired two-tailed t test, or the Wilcoxon signed rank test if indicated by a normality test (D'Agostino & Pearson ominibus normality test). Analysis were performed using GraphPad Prism version 5. As many statistical tests were carried out, the p<0.05 threshold was corrected for multiple testing: we referred to a Bonferroni-corrected p-value significance threshold of 0.05/24 = 0.002. P-values that are smaller than 0.05 but larger than 0.002 may be due to chance and do not infer the same strength of evidence as they would if a single test was carried out.

The stain index (SI) was calculated according to the formula SI  =  D/W, where D  =  difference between the medians of the positive and negative populations and W  =  spread (2×rSD) of the negative population [20].

### Computational analysis

The details of the methodology are described elsewhere [19], [21], [22]. Briefly, the flowType pipeline was used to identify cell populations, and the immunophenotypes with high area under the curve (AUC) score after a receiver operating characteristic (ROC) curve analysis were selected for analysis using RchyOptimyx [21], [23].

#### Terms and Definitions

A phenotype is the number of cells in a cell population divided by the total number of live T-cells.

A true positive (TP) is a Lyoplate sample that is correctly marked as Lyoplate. A false positive (FP) is a Liquid sample that is marked as Lyoplate by mistake. False negative (FN) and true negative (TN) are defined similarly.

Sensitivity measures the proportion of actual positives which are correctly identified as such (TP/TP+FN).

Specificity measures the proportion of actual negatives which are correctly identified as such (TN/TN+FP).

Accuracy measures the proportion of true results to all predictions ( TP+TN/FN+FP).

ROC Analysis: A phenotype can be thresholded to divide the subjects to positives and negatives. This threshold controls the trade-off between sensitivity and specificity. A ROC curve demonstrates different values of sensitivity and 1 – specificity that are obtained by changing this threshold. The AUC can be used as a measure of the predictive power of the phenotype. AUC is between 0,5 and 1 with 1 referring to a perfect phenotype and 0,5 to a random prediction.

Replication cohort: Six additional PBMC samples from healthy volunteers (four males and two females, mean age 34 years, range 23–43 years) were run as an independent cohort following the same experimental steps described for the principal study cohort.

## Results

### Flow Cytometry antibody cocktail and lyoplate design

Our goal was to assess the performance of a lyoplate-based flow cytometry platform (LFP) versus a conventional (liquid) flow cytometry platform (CFP) using a panel of 12 parameters (nine markers + one viability dye + FSC-A and SSC-A), including surface markers (CD3, CD4, CD8, CD45RO, and CD25) and intracellular markers (IFN-γ, IL-10, IL-17A, and Foxp3) focussing on the investigation of T cell subsets [T helper (Th) 1, Th17, T regulatory cells (T regs) and CD8^+^ T cells]. For induction of cytokine production, experiments were performed in the presence and absence of a polyclonal stimulation.

Antibody panel (Fig. S1A) was chosen after testing different antibody-fluorochrome combinations, maximizing antigen detection and minimizing major spectral overlaps between fluorochromes using FMO controls (data not shown). Lyoplate layout (Fig. S1B) was designed leaving empty wells between different samples and stimulation conditions to avoid cross contamination.

### Lyoplate based flow cytometry has higher sensitivity for IFN-γ and IL-10 detection than conventional flow cytometry

To quantitatively compare conventional and lyoplate-based flow cytometry platform (CFP and LFP respectively), peripheral blood mononuclear cells (PBMC) from healthy donors were stimulated and stained in parallel using liquid and lyophilized reagents (Fig. S1C). CFP and LFP derived bi-dimensional dot plots were similar (Fig. S2).

**Figure 1:**
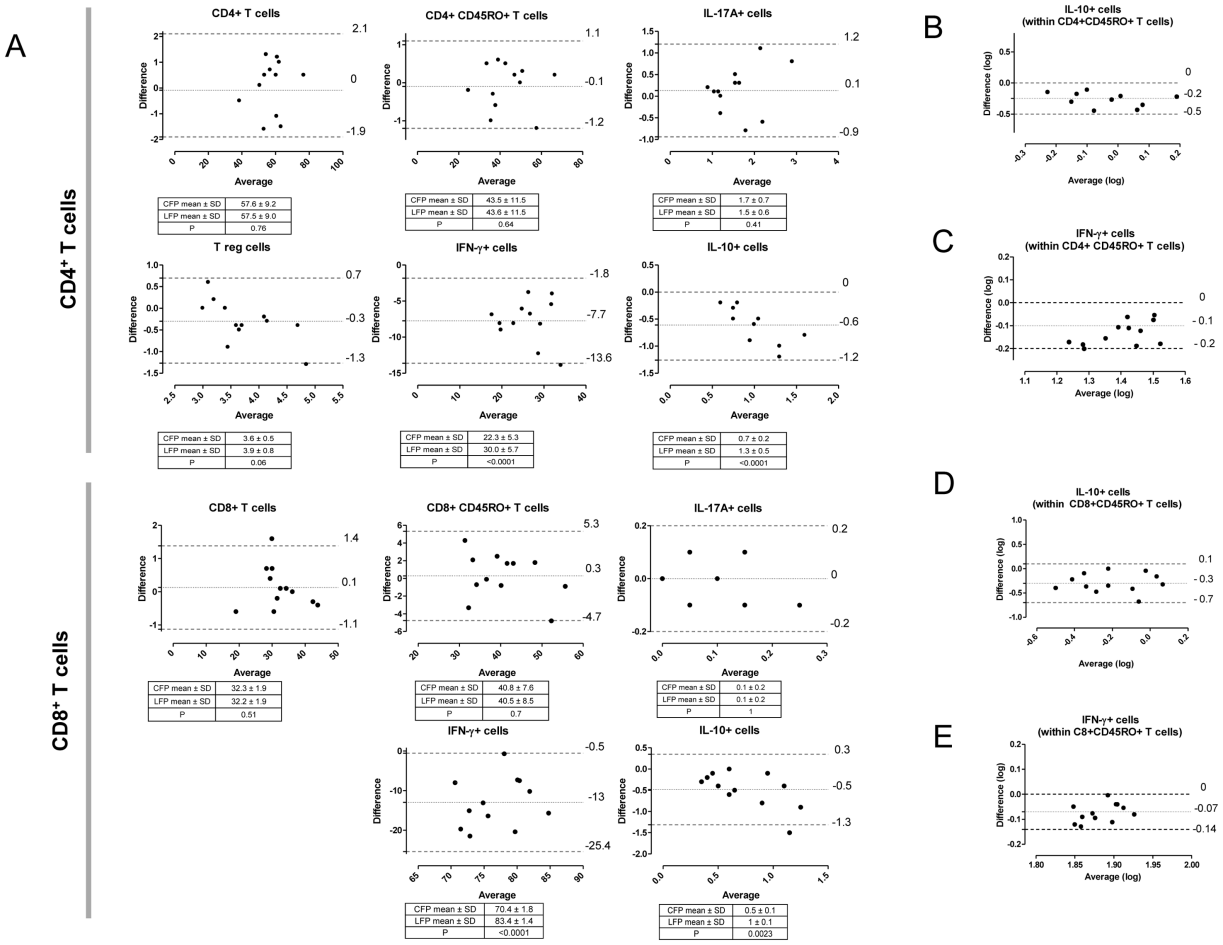
Lyoplate based flow cytometry has higher sensitivity for IFN-γ and IL-10 detection than conventional flow cytometry. **A**. Lyoplate based flow cytometry platform (LFP) results in increased detection of IFN-γ^+^ and IL-10^+^ cells compared to conventional (liquid) flow cytometry platform (CFP). Peripheral blood mononuclear cells (PBMC) from 12 healthy donors were incubated with (stimulated samples) or without (unstimulated samples) phorbol 12-myristate 13-acetate (PMA)/ionomycin in the presence of brefeldin A and monensin, either in the liquid or lyophilized form. Cells were then stained with liquid or lyophilized antibodies. Frequencies of CD3^+^CD4^+^, CD3^+^CD8^+^, CD3^+^CD4^+^CD45RO^+^ (memory CD4+ T cells), CD3^+^CD8^+^CD45RO^+^ (memory CD8+ T cells) and Tregs (CD3^+^CD4^+^CD25^high^FoxP3^+^) cells were calculated using unstimulated samples, while frequencies of IFN-γ^+^, IL-17A^+^, or IL-10^+^ cells were calculated from stimulated samples within memory CD4+ or memory CD8+ T cells. Cell frequencies obtained by LFP and CFP were compared using Bland Altman plots in which the differences between the cell frequencies obtained by the two methods (calculated as CFP minus LFP, y axis) are plotted against the cell frequency averages of the two methods (x axis). Horizontal lines are drawn at the mean difference (bias), and at the limits of agreement, which are defined as the mean difference ±1.96 times the SD of the differences. The tables associated with each plot indicate cell frequency mean ± SD measured by CFP and LFP, and the p value for a paired t test or Wilcoxon signed rank test, **P<0.01, ***P<0.001. **B-E.** IL-10+ (B,D) and IFN-γ^+^(C,E) cells, within CD4+ and CD8+ memory T cells, are plotted after logarithmic transformation.

Cell frequencies of the main T cell subsets and cytokine producing cells obtained by CFP and LFP were compared (Fig. 1). Both techniques showed comparable results for the detection of CD4^+^ T cells, CD8^+^ T cells, memory CD4^+^ T cells (identified as live CD3^+^CD4^+^CD45RO^+^ cells), memory CD8^+^ T cells (identified as live CD3^+^CD8^+^CD45RO^+^ cells), and Tregs (identified as live CD3^+^CD4^+^CD25^high^Foxp3^+^ cells), as shown by the small bias and relatively narrow 95% limits of agreement in the Bland-Altman plot (Fig. 1A). PBMC were stimulated with Phorbol 12-myristate 13-acetate (PMA)/Ionomycin and cytokine (IFN-γ, IL-10 and IL-17A) production assessed in memory CD4^+^ and CD8^+^ T cells (Fig. 1A).

LPF detected a higher frequency of IFN-γ^+^ and IL-10^+^ cells compared to CFP (Fig. 1A), as indicated by the dots below the bias line in the Bland Altman plots and the significance of paired t test.

For IL-10+ the systematic bias between the two methods was larger at higher frequencies (Fig 1A). After log-transformation, the bias and variability of the differences were more even across the frequency range (Fig 1 B, D), indicating that the size of the bias could be summarised as a percentage of the frequency of one method compared to the other. The frequencies of the CFP method were on average 44% and 49% (in CD4+ and C8+ memory T cell groups, respectively) lower than those from the LFP method. For IFN-γ^+^, (Fig. C, E), log-transformation led to more even variability in the differences across the frequency range, and an estimate that on average the frequencies from the CFP method were reduced by 15% and 26% (in CD8+ and C4+ memory T cell groups, respectively) compared to LFP.

We next investigated which component of the LFP (either the stimulation or the stain plate) was responsible for the increased detection of IFN-γ^+^ and IL-10^+^ cells. Results shown in Fig. S3 suggest that both a more effective cell stimulation and staining with lyophilized reagents contribute to an increased detection of markers such as IFN-γ and IL-10.

Taken together these results suggest that LFP has higher sensitivity to detect key cytokines.

### Lyophilized antibodies result in higher stain index in stained cells compared to liquid antibodies

Next, we qualitatively compared the performance of liquid and lyophilized antibodies by calculating the stain index (SI) of stained cells, which is a measure of the brightness of a marker and its resolution sensitivity with respect to a given optical configuration on a flow cytometer.

As shown in Table 1, displaying the mean SI ± SEM of cells stained with different antibodies, seven out of nine of the lyophilized antibodies showed a higher SI compared to the liquid antibodies (for example: Foxp3 Alexa Fluor 488, liquid SI  = 3.7, lyophilized SI  = 5.1) The SI of cells stained with two lyophilized antibodies conjugated with tandem dyes (APC-H7 and PE-Cy5) was lower compared to the cells stained with liquid antibodies, suggesting that these tandem fluorochromes might be more sensitive to the lyophilisation procedure.

**Table 1:** Stain indexes (SI) of cells stained with lyophilized antibodies were higher than the liquid counterparts.

Marker	Fluorochrome	SI Liquid antibody (mean±SEM)	SI Lyophilized antibody (mean±S\llpEM)	p value
CD3	APC-H7	6.3±0.5	5.8±0.4	ns
CD4	APC	22.7±1.3	26.9±1.31	***
CD8	BD Horizon V500	12.7±0.7	15.5±0.9	***
CD45RO	PE-Cy5	9.6±0.4	7.3±0.8	*
CD25	PE-Cy7	2.1±0.1	2.3±0.1	*
Foxp3	Alexa Fluor 488	3.7±0.1	5.1±0.2	***
IFN-γ	Alexa Fluor 700	34.2±2.2	35.6±2.8	ns
IL-17A	BD Horizon V450	32.8±1.1	38.4±3.6	ns
IL-10	PE	6.3±0.3	8.0±0.8	ns

Stain index, calculated as D/W, where D  =  difference between the medians of the positive and negative populations and W  =  spread (2× rSD) of the negative population, is indicated for each antibody-fluorochrome combination ± SEM. The SI was calculated at one time point. Paired t test or Wilcoxon signed rank test was performed, *P<0.05, ***P<0.001.

Overall these results suggest that the antibody-fluorochrome combinations used for the cocktail in this study are stable and perform better in a lyophilized format.

### Conventional and lyoplate-based flow cytometry platforms have comparable intra- and inter-assay variability

**Figure 2:**
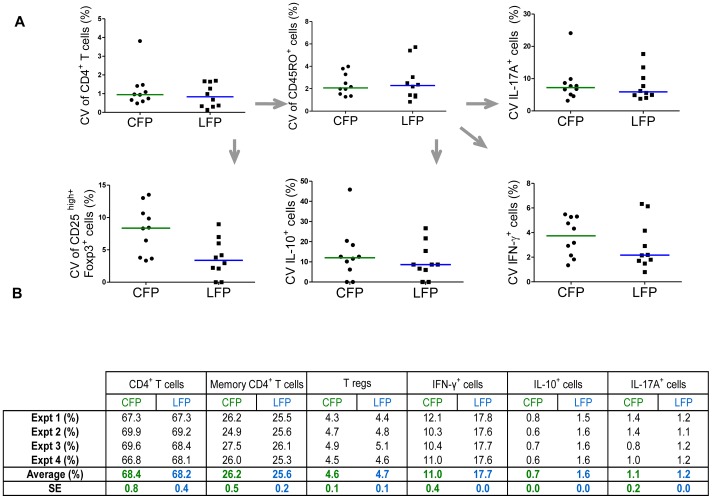
Conventional and lyoplate based flow cytometry platforms have comparable intra- and inter-assay variability. **A.** Comparison of intra-assay variability between conventional- and lyoplate based- flow cytometry platform (CFP and LFP respectively). Coefficient of variation (CV) was calculated for each sample from experimental triplicates at one time point. Arrows indicate the origin of daughter cell populations. Each dot corresponds to one individual, horizontal bars represent medians. **B.** Comparison of inter-assay variability between CFP and LFP. Cell frequencies obtained from the same leucocyte cone sample run across four different experiments. Percentages of IFN-γ^+^, IL-10^+^, and IL-17A^+^ cells were calculated within memory CD4^+^ T cells (identified as live CD3^+^CD4^+^CD45RO^+^ cells). T regs were identified as live CD3^+^CD4^+^CD25^high^Foxp3^+^ cells. Average and standard error (ER) are indicated in the two bottom rows.

In an effort to assess the reproducibility and robustness of CFP and LFP based experiments, we tested intra-assay variability in both settings by running each sample in triplicate and calculating the coefficient of variation (CV). As shown by the descriptive statistics in Fig. 2A, the average CVs for different cell populations were similar, reflecting comparable intra-assay variability between LFP and CFP. The CV of Tregs frequencies was lower in LFP compared to CFP, suggesting that a more accurate identification of Tregs might be possible by using lyoplates. Additionally, we tested inter-assay variability using two control samples run across multiple experiments. Fig. 2B shows the results obtained from one control sample run across four different experiments: the percentages of six cell populations investigated were consistent across experiments. Furthermore, when CFP and LFP based results were compared, mean cell frequency, with the exception of IL-10^+^ and IFN-γ^+^ cells as in Fig. 1, were similar, as was the standard error (SE). We concluded that LFP and CFP have comparable inter-assay variability.

### Computational analysis identifies novel cell populations

High dimensional data generated by multicolour flow cytometry require an unbiased and rapid analysis, difficult to perform using the conventional manual gating strategy; a ten colour panel generates 1024 theoretical cell populations (2^10^) to be analysed in a bidimensional space. We have therefore applied a computational analysis pipeline approach to our dataset.

**Figure 3:**
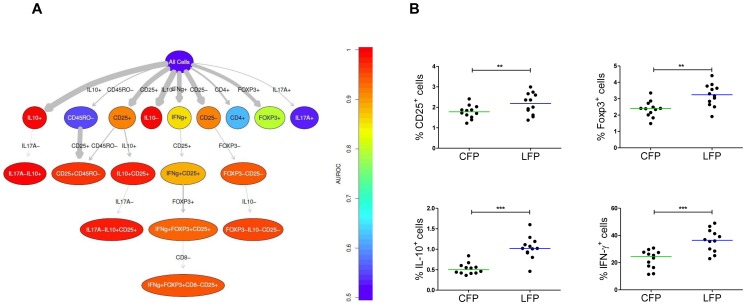
Computational analysis identifies novel cell populations. **A.** A cellular hierarchy for the selected immunophenotypes. The edge-to-edge width demonstrates the amount of predictive power (AUROC) gained by moving from one node to another. The color of the nodes demonstrates the predictive power of the cell population. This shows that IL-10, IFN-γ, CD25, and Foxp3 are the most discriminative markers; CD25 and Foxp3 were previously unidentified with conventional manual analysis. **B.** Manual analysis confirms differences obtained by computational analysis. Frequencies of CD25^+^, Foxp3^+^, IL-10^+^, and IFN-γ^+^ live T cells are increased in lyoplate based flow cytometry platform (LFP) compared to conventional flow cytometry platform (CFP) experiments. Each dot represents the average of experimental triplicates at two different time points. Paired t test or Wilcoxon signed rank test was performed, **P<0.01, ***P<0.001.

flowType [21] was used to extract 6560 cell populations from every FCS file. flowMeans [19] was used as the population identification algorithm. The IL-10^+^, IL-17A^+^, and Foxp3^+^ populations were too small to be automatically identified. For these markers, all of the samples were combined into a single file to allow a more robust population identification using a static gate. The measured immunophenotypes were analyzed using receiver operator characteristic (ROC) curves. A cumulative distribution function (CDF) of the area under the curve (AUC) values is illustrated in Figure S4A. The immunophenotypes with an AUC score of higher than 0.9 were selected for analysis using RchyOptimyx [23] (Fig. 3A). To include all of the single-marker immunophenotypes, the CD4^+^, Foxp3^+^, IL-17A^+^, and CD8^+^ cell populations were manually added to the results despite their low score. Fig. 3A shows that detection of IL-10, CD25, IFN-γ, and Foxp3 positive cells differs between liquid and lyoplates. While differences in the detection of IL-10^+^ and IFN-γ^+^ cells were already identified by manual analysis, differences in CD25^+^ and Foxp3^+^ cells are uniquely discovered by computational analysis. These results are confirmed by the ROC curves in Figure S4B. To confirm these findings, similar analysis was repeated in a validation cohort using new samples. The results of the ROC analysis in Figure S4C confirm the predictive power of IL-10, CD25, and Foxp3, while IFN-γ was not confirmed in the validation set.

A manual analysis, using the gating strategy shown in Fig. S5, confirmed the results obtained by computational analysis (Fig. 3B).

Thus, computational analysis reliably analyses flow cytometry data and identifies cell populations otherwise undetected by conventional manual analysis.

## Discussion

In this study we compared the performance of a conventional (liquid, CFP) versus a lyoplate-based flow cytometry platform (LFP) and the potential to integrate flow cytometry with computational data analysis to establish a robust framework to conduct biomarker discovery studies in humans.

We found that LFP has a higher sensitivity for detecting key cytokines (IFN-γ, IL-10) and activation markers (CD25, Foxp3) compared to CFP, while keeping comparable intra- and inter-assay variability.

Moreover, when computational analysis was performed by using RchyOptimix, novel immunophenotypes were identified.

Multicolour flow cytometry is becoming a preferential tool for immuno-monitoring and biomarker discovery in large human studies, thus requiring standardization of both experimental and analytical methods. However, available data refer to relatively small antibody cocktails and include the most common fluorochromes, thus only allowing the detection of a restricted set of markers [13], [15], [16].

Here, we measured for the first time 12 parameters using a LFP with and without polyclonal cell activation. Its positive performance for immunophenotyping and cytokine detection makes it a suitable alternative to CFP.

LFP has the advantage of simplifying the experimental protocol, is time saving (∼3 hours in each of our experiments), and allows a medium-throughput processing of the samples, using pre-filled 96 well plates and a plate loader. Moreover, pre-formatted lyoplates, containing the same batch of reagents, can be reliably used through the entire duration of a study and across multiple centres. Therefore, LFP reduces hands-on time, while promoting automation and reagent standardization that are of primary importance in translational and clinical research studies.

Our data indicate that lyophilized reagents resulted in more powerful cell stimulation and better marker discrimination, possibly due to improved reagent stability after lyophilisation. In keeping with the increased detection of IFN-γ^+^, IL-10^+^, Foxp3^+^ and CD25^+^ cells, most of the lyophilized antibodies also resulted in increased resolution sensitivity as determined by a higher stain index (SI) on stained PBMC. Of note, tandem dyes PE-Cy5 and APC-H7 showed a decreased SI on stained PBMC compared to cells stained with liquid counterparts, indicating that lyophilisation might have a different impact on different fluorochromes. This aspect should be considered when designing the antibody cocktail to be lyophilized, and a pre-test of the lyophilisation impact onto the specific antibody-fluorochrome combinations should be performed, especially for tandem dye conjugates. If possible, choosing an antibody batch with the brightest SI on stained cells could further help balance the lyophilisation effect.

Reproducible results, a key aspect in multicenter trials, require minimal intra- and inter-assay variability. In order to reduce assay variation, we combined the use of lyoplates with strict SOPs for sample handling, rigorous instrument QC, and reproducible instrument setup. CFP and LFP showed minimal intra-assay variability, suggesting that experimental replicates are not an absolute requirement for flow cytometry analysis. Importantly, LFP allowed a more accurate detection of Tregs. This is relevant as Tregs gating is notoriously difficult and subjective [24].

The high dimensional data generated by multi-parameter cell analysis need to be analysed in an unsupervised, multidimensional, and fast manner to overcome the subjectivity and non-reproducibility of manual gating and analysis. In recent years, several computational tools for analysis of flow cytometry data have been developed by different research groups (see [25] for a review). Two broad categories of these tools have recently been evaluated by the FlowCAP project [26]:(1) Clustering algorithms for automated identification of cell populations (e.g., [19], [27], [28]) and (2) Binary sample classification pipelines for identification of immunophenotypic differences between two groups of samples (e.g., [21], [29], [30]). The pipeline used in this work has been designed for identification of cell populations that correlate with an external variable (e.g., a clinical outcome). Detailed descriptions are available elsewhere [19], [21], [23]. Briefly, the pipeline can incorporate the background knowledge of the human experts into the gating process. Then, tens of thousands of immunophenotypes extracted from each sample are tested for correlation with the external variable (in this case, 6560 cell populations from every FCS file were correlated with the reagent type). Finally, the selected immunophenotypes are organized in a hierarchical structure based on their most common parent populations. These hierarchies not only provide intuitive data visualization, but also aid in adjusting the trade-off between the number of markers included in identification of a cell population of interest and the statistical significance of the correlation with the external variable. This information can also help in the use of high-dimensional datasets to guide the design of low-dimensional panels: for example a Time-of-Flight mass spectrometer (CyTOF) assay on a small dataset can analyze a large list of candidate markers, and using the hierarchies produced by this approach one can design lyoplate panels for further validation of the results.

Taken together, we propose the integration of LFP and computational analysis as a robust and standardized method for obtaining high content information on T cell proportions and functions in a medium-to-high-throughput manner. A natural application of this approach would be in the biomarker discovery arena, where the easy scalability of LFP with unbiased automated data analysis would allow the rapid and standardized screening of large human cohorts.

## Supporting Information

Figure S1 **Lyoplate design and experimental workflow.** **A.** Flow cytometry antibody cocktail. For each antibody, the antigen specificity, the conjugated fluorochrome, and the clone is indicated. **B.** Lyoplate layout. Yellow and orange wells contain peripheral blood mononuclear cells (PBMC) from healthy volunteers, while grey wells are dedicated to inter-assay controls: either Streck CD Check Plus cells or cells from a leucocyte cone (LC). CON (yellow and light grey) indicate unstimulated control samples (containing monensin and brefeldin A) while PMA/I (orange and dark grey) indicate wells containing phorbol 12-myristate 13-acetate (PMA)/ionomycin/monensin and brefeldin A. A, B and C indicate experimental triplicates. **C.** Flow cytometry experimental workflow using a conventional (liquid) flow cytometry platform (CFP, on the right) or a lyoplate-based flow cytometry platform (LFP, on the left).
(TIF)

Figure S2 **Comparison of dot plots generated by conventional- or lyoplate based- flow cytometry platform.** Representative dot plots of main T cell subsets and cytokine producing cells obtained by conventional (top panel) and lyoplate-based (bottom panel) flow cytometry platform. First, live CD3+ cells were selected, cell debris and doublets were excluded using FSC/SSC properties, and then populations of interest were selected. Cytokine producing cells were gated within memory CD4+ T cells (identified as live CD3+CD4+CD45RO+ cells). Arrows indicate the origin of daughter cell populations.
(TIF)

Figure S3 **Both lyoplate-based cell stimulation and staining contribute to an increased detection of cytokines and activation markers.** Samples were stimulated (with phorbol 12-myristate 13-acetate (PMA)/ionomycin/monensin and brefeldin A) and stained either with liquid (green boxplots) or lyophilized (blue boxplots) reagents, or were stimulated and stained in a mixed protocol, with liquid reagent-based stimulation and lyophilized reagent-based staining (grey boxplots) or vice versa (brown boxplots). Results show data from three independent experiments.
(TIF)

Figure S4 **Cumulative distribution function and receiver operating characteristic analysis.** **A.** The cumulative distribution function (CDF) of the area under the curve (AUC) values of all phenotypes. The phenotypes with high AUC scores were selected as candidate cell types that can discriminate between lyoplate based- (LFP) and conventional- (CFP) flow cytometry platform analyzed samples. The red dashed-line shows the current cut-off (0.9). **B.** Receiver operating characteristic (ROC) analysis of the single-marker phenotypes. IL-10, CD25, IFN-γ and Foxp3 were the discriminative markers between CFP and LFP based generated data. **C.** ROC analysis of the single-marker phenotypes in the validation cohort. IL-10, CD25 and Foxp3 confirmed their predictive power.
(TIF)

Figure S5 **Gating strategy for manual analysis to confirm automated analysis-derived results.** Starting from the top left dot plot, live CD3^+^ cells were selected, cell debris and doublets were excluded using FCS/SSC properties, and frequencies of Foxp3^+^, CD25^+^, IL-10^+^, and IFN-γ^+^ cells were derived.
(PDF)
