# Supplementary figures and images for: Integration of Lyoplate Based Flow Cytometry and Computational Analysis for Standardized Immunological Biomarker Discovery
Source: PLoS One. 2013 Jul 3;8(7):e65485. doi: 10.1371/journal.pone.0065485 (PMC3701052; doi:10.1371/journal.pone.0065485)

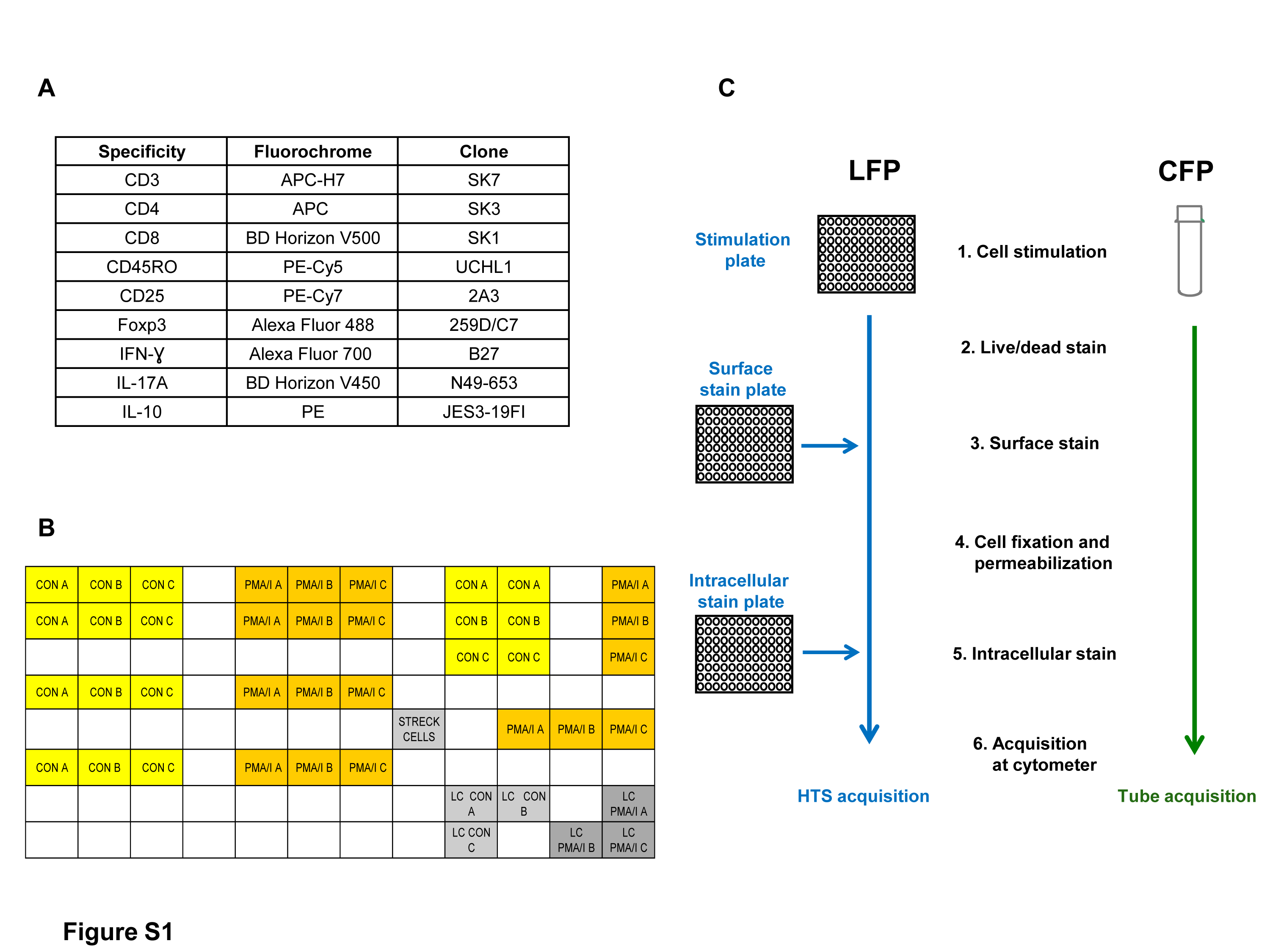

Supplement: Figure S1 — Lyoplate design and experimental workflow. A. Flow cytometry antibody cocktail. For each antibody, the antigen specificity, the conjugated fluorochrome, and the clone is indicated. B. Lyoplate layout. Yellow and orange wells contain peripheral blood mononuclear cells (PBMC) from healthy volunteers, while grey wells are dedicated to inter-assay controls: either Streck CD Check Plus cells or cells from a leucocyte cone (LC). CON (yellow and light grey) indicate unstimulated control samples (containing monensin and brefeldin A) while PMA/I (orange and dark grey) indicate wells containing phorbol 12-myristate 13-acetate (PMA)/ionomycin/monensin and brefeldin A. A, B and C indicate experimental triplicates. C. Flow cytometry experimental workflow using a conventional (liquid) flow cytometry platform (CFP, on the right) or a lyoplate-based flow cytometry platform (LFP, on the left). (TIF) [file pone.0065485.s001.tif]

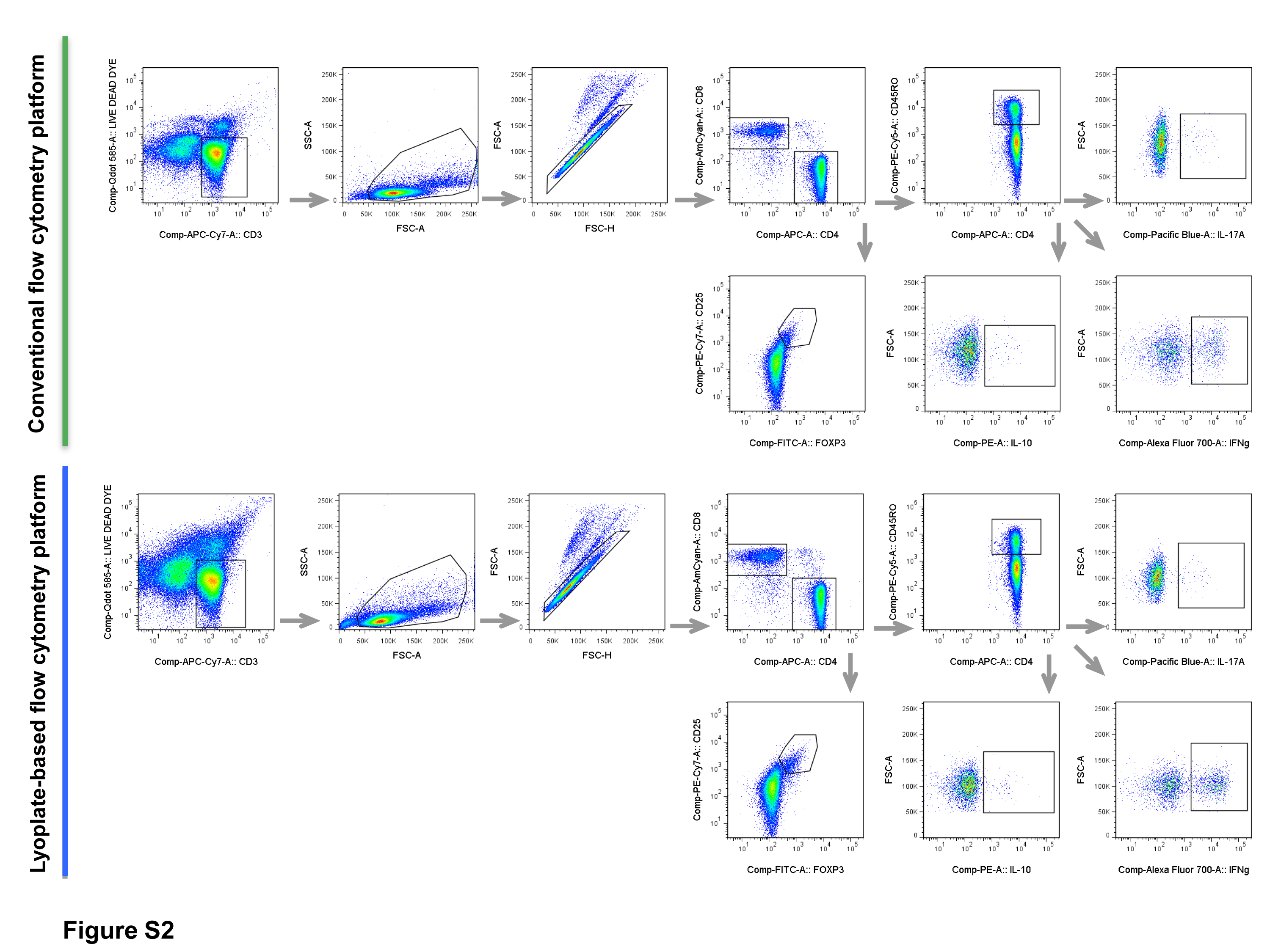

Supplement: Figure S2 — Comparison of dot plots generated by conventional- or lyoplate based- flow cytometry platform. Representative dot plots of main T cell subsets and cytokine producing cells obtained by conventional (top panel) and lyoplate-based (bottom panel) flow cytometry platform. First, live CD3+ cells were selected, cell debris and doublets were excluded using FSC/SSC properties, and then populations of interest were selected. Cytokine producing cells were gated within memory CD4+ T cells (identified as live CD3+CD4+CD45RO+ cells). Arrows indicate the origin of daughter cell populations. (TIF) [file pone.0065485.s002.tif]

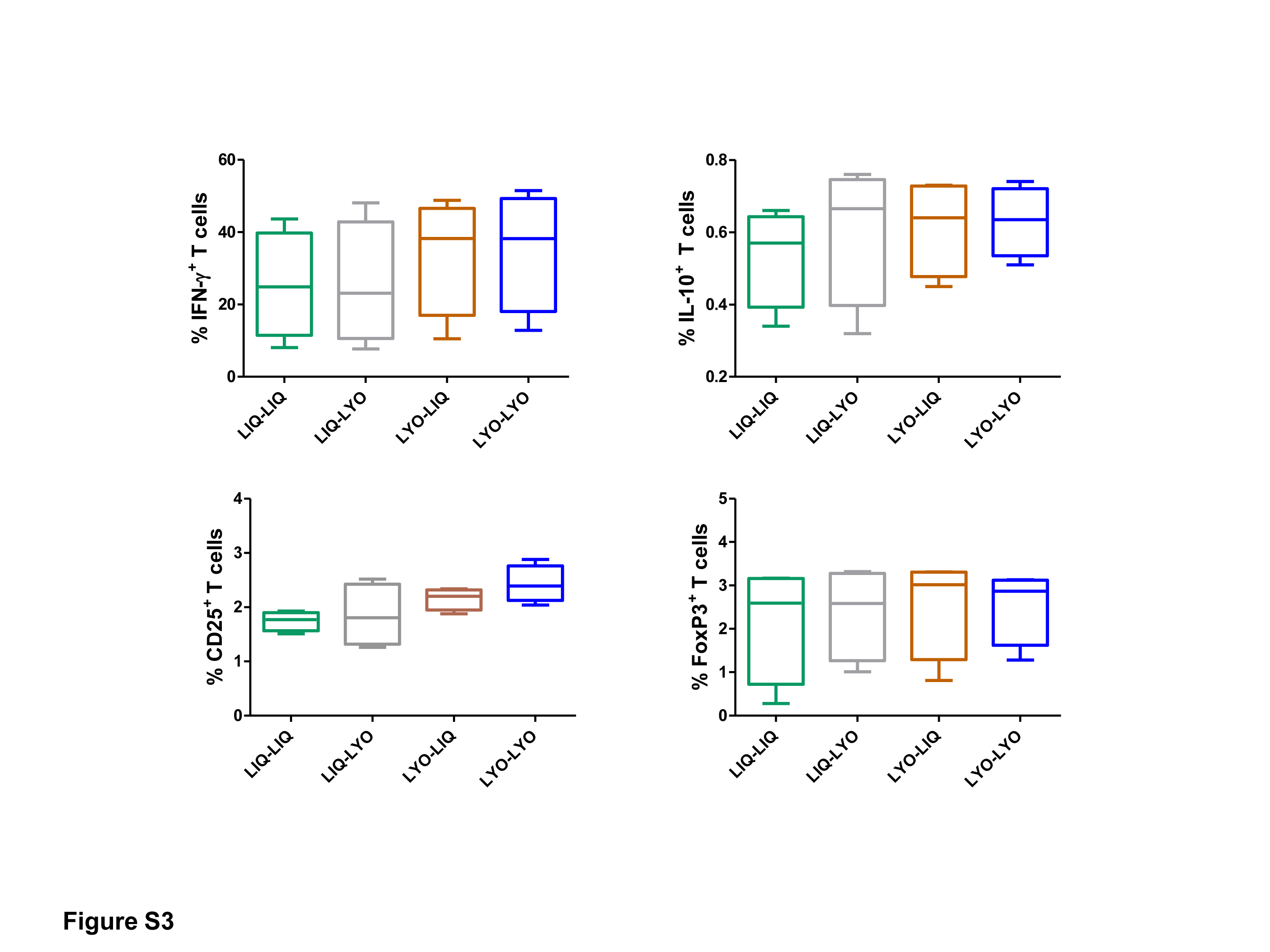

Supplement: Figure S3 — Both lyoplate-based cell stimulation and staining contribute to an increased detection of cytokines and activation markers. Samples were stimulated (with phorbol 12-myristate 13-acetate (PMA)/ionomycin/monensin and brefeldin A) and stained either with liquid (green boxplots) or lyophilized (blue boxplots) reagents, or were stimulated and stained in a mixed protocol, with liquid reagent-based stimulation and lyophilized reagent-based staining (grey boxplots) or vice versa (brown boxplots). Results show data from three independent experiments. (TIF) [file pone.0065485.s003.tif]

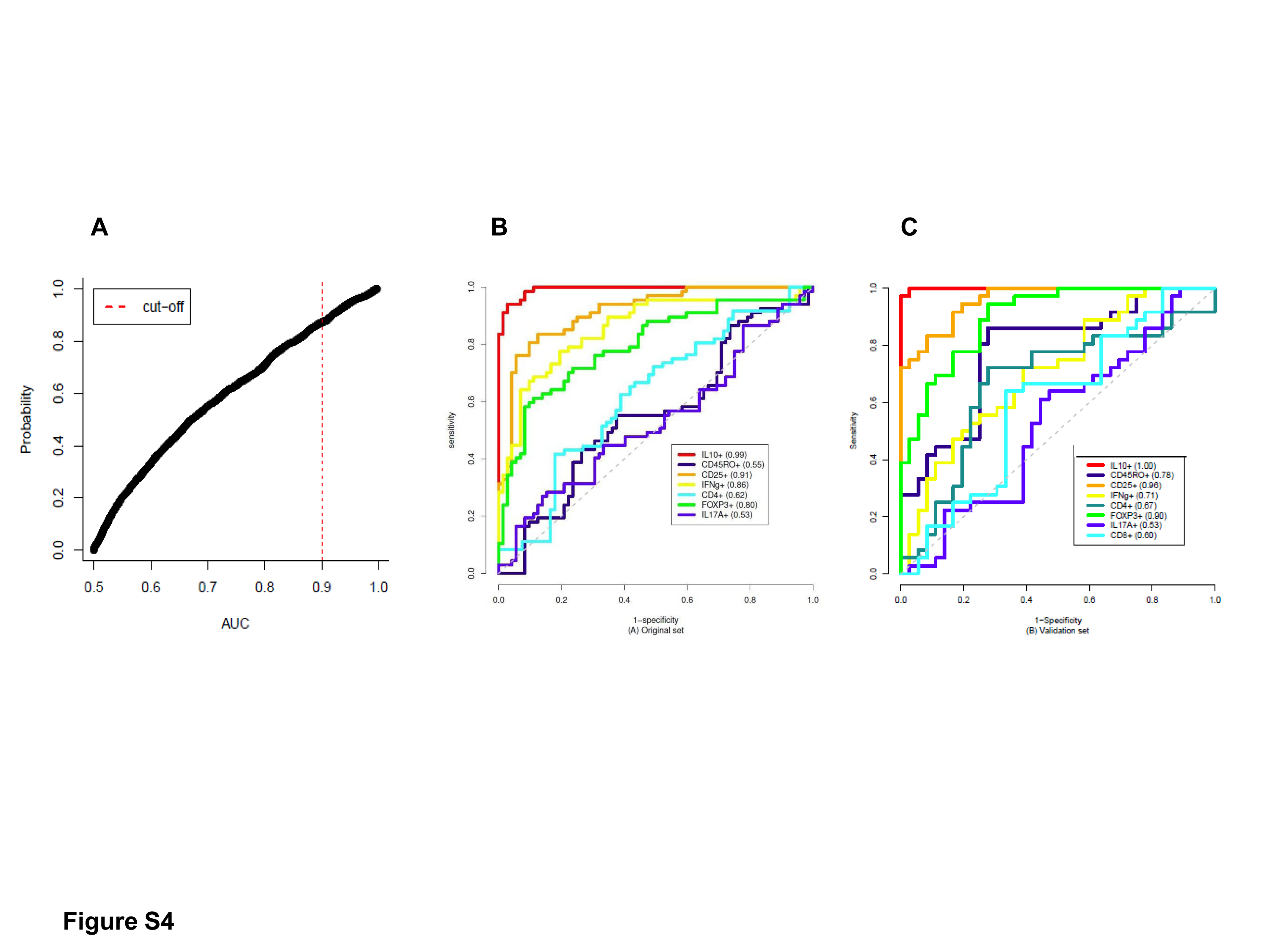

Supplement: Figure S4 — Cumulative distribution function and receiver operating characteristic analysis. A. The cumulative distribution function (CDF) of the area under the curve (AUC) values of all phenotypes. The phenotypes with high AUC scores were selected as candidate cell types that can discriminate between lyoplate based- (LFP) and conventional- (CFP) flow cytometry platform analyzed samples. The red dashed-line shows the current cut-off (0.9). B. Receiver operating characteristic (ROC) analysis of the single-marker phenotypes. IL-10, CD25, IFN-γ and Foxp3 were the discriminative markers between CFP and LFP based generated data. C. ROC analysis of the single-marker phenotypes in the validation cohort. IL-10, CD25 and Foxp3 confirmed their predictive power. (TIF) [file pone.0065485.s004.tif]

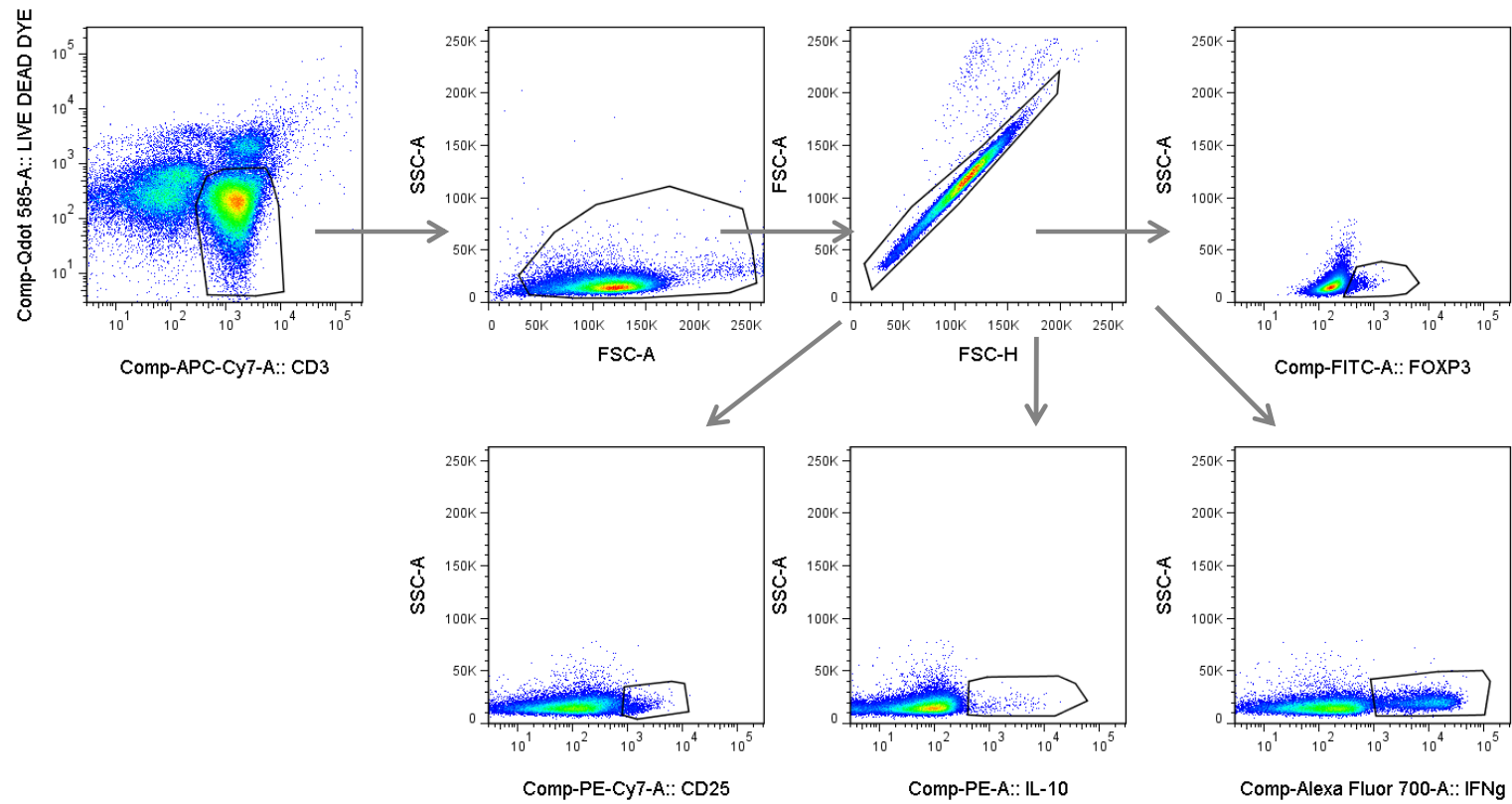

**Figure S5**

Supplement: Figure S5 — Gating strategy for manual analysis to confirm automated analysis-derived results. Starting from the top left dot plot, live CD3+ cells were selected, cell debris and doublets were excluded using FCS/SSC properties, and frequencies of Foxp3+, CD25+, IL-10+, and IFN-γ+ cells were derived. (PDF) [file pone.0065485.s005.pdf]
